# The Complete Chloroplast Genome of the Vulnerable *Oreocharis esquirolii* (Gesneriaceae): Structural Features, Comparative and Phylogenetic Analysis

**DOI:** 10.3390/plants9121692

**Published:** 2020-12-02

**Authors:** Li Gu, Ting Su, Ming-Tai An, Guo-Xiong Hu

**Affiliations:** 1 College of Life Sciences, Guizhou University, Guiyang 550025, China; guligz@163.com (L.G.); sutingyo@163.com (T.S.); 2 The Key Laboratory of Plant Resources Conservation and Germplasm Innovation in Mountainous Region Ministry of Education, Guizhou University, Guiyang 550025, China; 3 Institute of Agro-Bioengineering, Guizhou University, Guiyang 550025, China; 4 College of Forestry, Guizhou University, Guiyang 550025, China; mtan@gzu.edu.cn

**Keywords:** Gesneriaceae, next-generation sequencing, complete chloroplast genome, *Oreocharis*, *Thamnocharis*

## Abstract

*Oreocharis esquirolii*, a member of Gesneriaceae, is known as *Thamnocharis esquirolii*, which has been regarded a synonym of the former. The species is endemic to Guizhou, southwestern China, and is evaluated as vulnerable (VU) under the International Union for Conservation of Nature (IUCN) criteria. Until now, the sequence and genome information of *O. esquirolii* remains unknown. In this study, we assembled and characterized the complete chloroplast (cp) genome of *O. esquirolii* using Illumina sequencing data for the first time. The total length of the cp genome was 154,069 bp with a typical quadripartite structure consisting of a pair of inverted repeats (IRs) of 25,392 bp separated by a large single copy region (LSC) of 85,156 bp and a small single copy region (SSC) of18,129 bp. The genome comprised 114 unique genes with 80 protein-coding genes, 30 tRNA genes, and four rRNA genes. Thirty-one repeat sequences and 74 simple sequence repeats (SSRs) were identified. Genome alignment across five plastid genomes of Gesneriaceae indicated a high sequence similarity. Four highly variable sites (*rps16-trnQ*, *trnS-trnG*, *ndhF-rpl32*, and *ycf 1*) were identified. Phylogenetic analysis indicated that *O. esquirolii* grouped together with *O. mileensis*, supporting resurrection of the name *Oreocharis esquirolii* from *Thamnocharis* *esquirolii*. The complete cp genome sequence will contribute to further studies in molecular identification, genetic diversity, and phylogeny.

## 1. Introduction

Traditionally, *Oreocharis* Benth. was a genus of the Gesneriaceae including 27 species [1,2]. Phylogenetic researches showed that *Oreocharis* was not monophyletic and up to 10 other genera were transferred to the genus [3,4,5]. Recently, an increasing number of new species of *Oreocharis* have been discovered and now approximately 135 species are recorded within this genus [6,7]. *Oreocharis* is mainly distributed in the tropical and subtropical areas in the south and southwest of China with a few extending to neighboring countries, such as Vietnam, Thailand, and Japan [3,8,9,10,11].

*Oreocharis esquirolii* H. Lév. was first established by Augustin Abel Hector Léveillé in 1911 based on a collection (*Esquirol 628*) from Guizhou, southwestern China [12]. Based on the character of actinomorphic corolla, Wang [13] transferred this species to his newly established genus, namely *Thamnocharis esquirolii* (H. Lév.) W. T. Wang. However, molecular phylogenetic results showed that *Thamnocharis* was embedded into *Oreocharis* [3,4], and Möller et al. [4] resurrected *Oreocharis esquirolii* with regarding *Thamnocharis* as a synonym of *Oreocharis*.

*Oreocharis esquirolii* is endemic to Zhenfeng and Xingren County, Guizhou, southwestern China with a narrow distribution [2]. The species grows in thicket or hilly forest at an altitude of about 1500–1600 m. Due to excessive deforestation, serious vegetation damage and habitat degradation or loss, the population of the species decreased significantly with a risk of extinction. Based on restriction in habitat coupled with other threats, *O. esquirolii* was evaluated as vulnerable (VU) in IUCN’s Red List of Threatened Species (http://www.iucnredlist.org/) and was listed as a national grade-I protected plant by China’s government in 1999. 

In plants, chloroplast (cp) genome is highly conserved in gene order, gene content, and genome organization [14,15] with a typical quadripartite structure consisting of a large single copy region (LSC), a small single-copy region (SSC), and a pair of inverted repeats (IRs) [16,17]. In most angiosperm chloroplasts, the cp genome ranges from 72 kb to 217 kb [18]. Chloroplast genome usually codes for 110–130 genes, including about 80 protein-coding genes, four rRNA genes, and about 30 tRNA genes [19]. The highly conserved structure of cp genome makes it often used to infer evolutionary relationships of higher taxa [20]. Currently, cp genome has also been demonstrated to be effective to solve species-level phylogenetic relationships in some taxa [21,22,23]. Comparative analysis of cp genome can provide valuable information for understanding structural and organizational changes of plant cp genome, and effectively help to reveal processes of plant molecular evolution and diversification [16,24,25]. Although cp genomes of some taxa of Gesneriaceae have been reported [26,27,28,29], the cp genome of *O. esquirolii* is not included. In this study, we sequenced the cp genome of *O. esquirolii* for the first time, aiming to present the genomic features of *O. esquirolii* and compare its structure and gene organization within Gesneriaceae. In addition, based on available cp genomes in GenBank, we inferred its phylogenetic position in Gesneriaceae.

## 2. Results and Discussion

### 2.1. Gene Content and Structure of Chloroplast Genome of Oreocharis esquirolii

**Figure 1:**
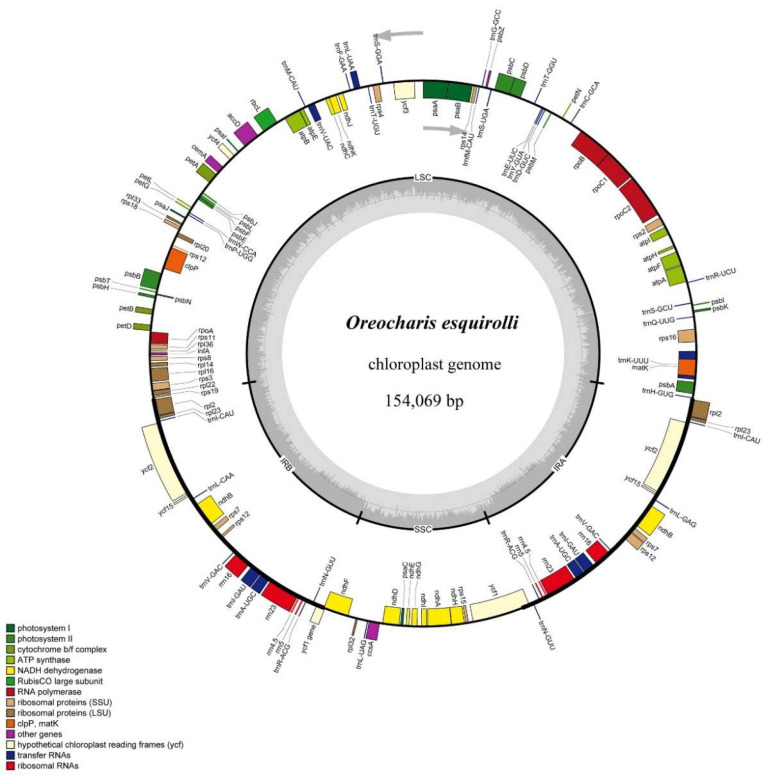
Gene map of chloroplast genome of *Oreocharis esquirolii*. Genes outside the circle are transcribed in counterclockwise direction and those inside in clockwise direction. LSC indicates large single copy; SSC small single copy, and IR inverted repeat.

Generally, the angiosperm cp genome is considered to be conserved [30]. In this study, we sequenced the cp genome of *Oreocharis esquirolii* and compared its features with other species from Gesneriaceae. The cp genome features of *O. esquirolii* were similar to other reported species in the Gesneriaceae concerning gene content, order, and orientation [28,31]. The whole cp genome of *O. esquirolii* was found to be 154,069 bp in length with a typical quadripartite structure, comprising a pair of inverted repeats (IRa and IRb) of 25,392 bp separated by a LSC region of 85,156 bp and a SSC region of 18,129 bp (Figure 1). Additionally, comparisons of length and GC content with the other 16 species from Gesneriaceae showed that their lengths ranged from 152,373 bp (*Primulina eburnea*) to 154,069 bp (*O. esquirolii*) and the GC content from 37.40% (*O. mileensis*) to 37.59% (*Primulina huaijiensis*) (Table S1). Notably, *O. esquirolii*, has the longest overall length (154,069 bp) but the shortest IR regions (25,392 bp), which may be related to the contraction of the IR regions.

Gene annotation revealed that the cp genome of *O. esquirolii* contained 114 unique genes, including a set of 80 protein-coding genes, 30 tRNA genes, and four rRNA genes. Amongst them, 19 genes were duplicated in the IR regions, comprising eight protein-coding genes (*ndhB*, *ycf1*, *ycf2*, *ycf15*, *rpl2*, *rps7*, *rpl23*, and *rps12*), four rRNA genes (*rrn4.5*, *rrn23*, *rrn5*, and *rrn16*), and seven tRNA genes (*trnA^-UGC^*, *trnI^-CAU^*, *trnI^-GAU^*, *trnL^-CAA^*, *trnN^-GUU^*, *trnV^-GAC^*, and *trnR^-ACG^*) (Table 1). Fourteen intron-containing genes were detected, including nine protein-coding genes (*atpF*, *ndhA*, *ndhB*, *rpl2*, *rpl16*, *rps16*, *clpP*, *rpoC1*, and *ycf3*) and five tRNA genes (*trnA^-UGC^*, *trnI^-GAU^*, *trnK^-UUU^*, *trnL^-UAA^*, and *trnV^-UAC^*). Of the 14 genes, two (*clpP* and *ycf3*) harbored two introns and the other 12 contained only one intron with the *trnK^-UUU^* including the largest intron (2,497 bp) and the *trnL^-UAA^* having the smallest intron (476 bp) (Table 2). Content (%) of the four bases was T (31.67%) > A (30.83%) > C (19.04%) > G (18.45%). Similarly to previous reports [26,30], the GC content in the IR regions of *O. esquirolii* (43.21%) was higher than that in the LSC (35.43%) and SSC (31.16%) (Table 3), which could be attributed to the presence of the eight rRNA sequences in IR regions [32].

### 2.2. Codon Usage Bias Analysis

**Figure 2:**
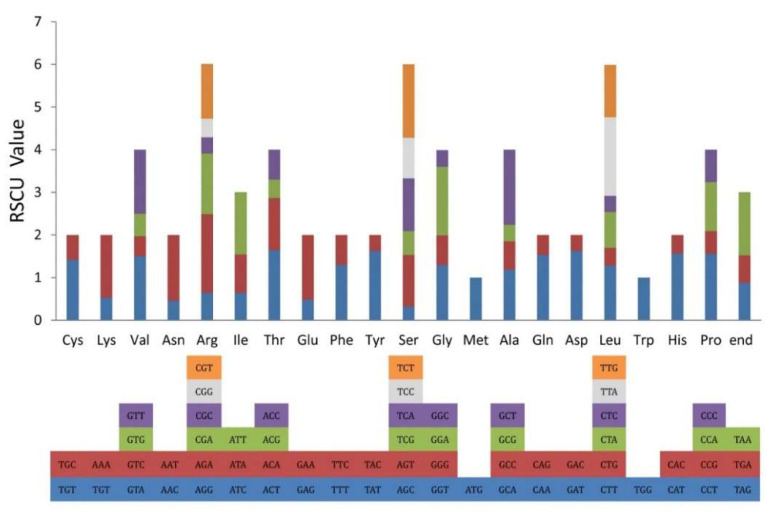
Amino acid frequencies and RSCU value of the protein-coding sequences of *Oreocharis esquirolii*.

Codon usage refers to an organism’s use of similar codons when encoding amino acids. Non-random use of synonymous codons is widespread both within and between organisms [33]. Many studies have shown that there are species-specific patterns of codon usage due to various factors such as codon hydrophilicity, gene length, expression levels, and protein secondary structure base composition [34,35]. The frequency of codons in the cp genome of *Oreocharis esquirolii* was calculated based on protein-coding genes. In total, all genes were encoded by 26,550 codons, of which AUU (Ile) was the most frequent (1111 codons) and UGC (Cys) was the least frequent (90 codons). Among the amino acids encoded by these codons, Leucine (2,784 codons, 10.49%), with the highest coding rate, was the most frequent. However, Cysteine (309 codons, 1.16%) was found less due to their high sensitivity to changes in physiological and environmental conditions [36] (Table S2). If the relative value of synonymous codon usage (RSCU) is greater than one, the codon usage is highly preferred, indicating that the codon is used more often than expected but not preferred if the value is equal to one and less preferred with values of less than one [36,37]. Codon usage analysis showed that codon usage was biased towards T and A at the third codon position in the cp genome of *O. esquirolii*. Furthermore, 30 highly preferred codons were detected in the *O. esquirolii* with an RSCU value greater than 1.0. Of the 30 codons, except for UUG ending with G, all codons terminated with A or T, and no C was found in the third position (Figure 2, Table S2).

### 2.3. SSRs Analysis

**Figure 3:**
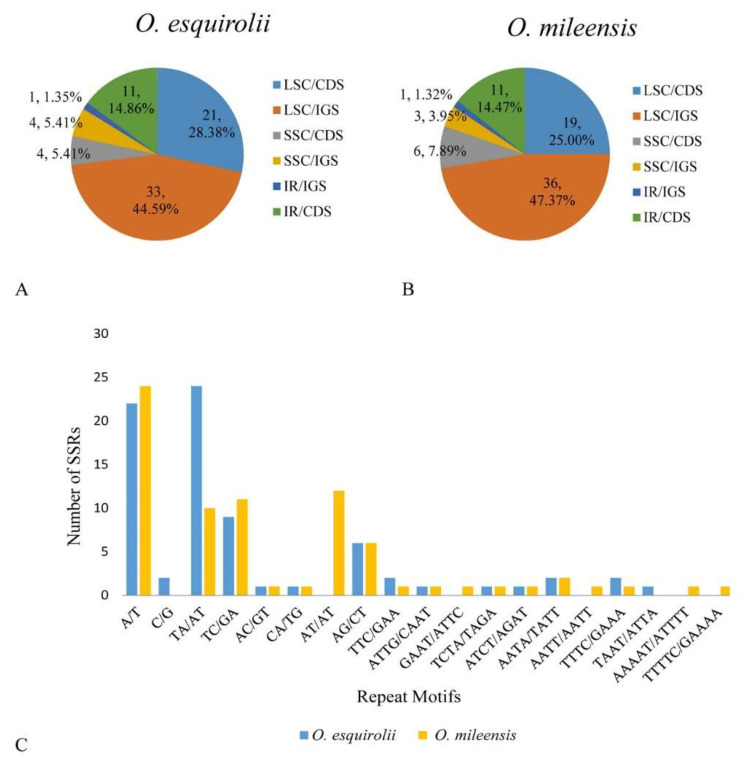
Simple Sequence Repeats (SSRs) in cp genomes of *Oreocharis esquirolii* and *O. mileensis*. (**A**,**B**) Frequencies of identified SSRs in LSC, IR, and SSC regions; (**C**) Numbers of SSRs.

Simple sequence repeats (SSRs) are tandemly repeats of DNA sequences, comprising one to six (mono-, di-, tri-, tetra-, penta-, and hexa-) repeat nucleotide units. Being highly reliable, reproducible, and highly polymorphic, SSRs have been widely applied in molecular identification, genetic diversity, and population genetic studies [22,38,39,40]. In this study, SSRs of both *Oreocharis esquirolii* and *O. mileensis* were analyzed. A total of 74 SSRs were found in *O. esquirolii*, of which 54 were in the LSC regions, 12 in the IR and eight in the SSC regions. Comparatively, in the congeneric *O. mileensis*, 76 SSRs were detected with 55, 12, and nine SSRs distributed in the LSC, IR, and SSC regions, respectively (Figure 3). Besides, 27 SSRs were discovered in the coding sequences (CDS), 38 in the intergenic spacers (IGS), and nine in the intron regions of the *O. esquirolii* cp genome, whereas the values in the *O. mileensis* were 29 in CDS, 38 in IGS and nine in intron regions (Table S3). In terms of repeat unit, total five types of repeats (mono-, di-, tri-, tetra-, and penta-) were detected in *O. esquirolii* and *O. mileensis* cp genomes. Dinucleotide repeats were the most frequent, accounting for 55.41% (41) and 53.95% (41), respectively, followed by mononucleotide with 32.43% (24) and 31.98% (24), tetranucleotide with 10.81% (8) and 10.53% (8), and the least frequent trinucleotide with 1.35% (1) and 1.32% (1). It is worth noting that the pentanucleotide repeats (2, 2.63%) were only detected in *O. mileensis*, (Figure 3A,B, Table S3). Among the identified repeat units, dinucleotide repeat unit (AG/CT and AT/TA) was the most abundant. This finding supports the view that cp SSRs are generally composed of short polyadenine (polyA) or polythymine (polyT) repeats and rarely contain tandem guanine (G) or cytosine (C) repeats [40,41]. In addition, rarity or absence of pentanucleotide and hexanucleotide repeats in these two species demonstrated again that the two types of repeat unit are rather rare among cp SSRs [26,40].

### 2.4. Analysis of Repeat Sequences

**Figure 4:**
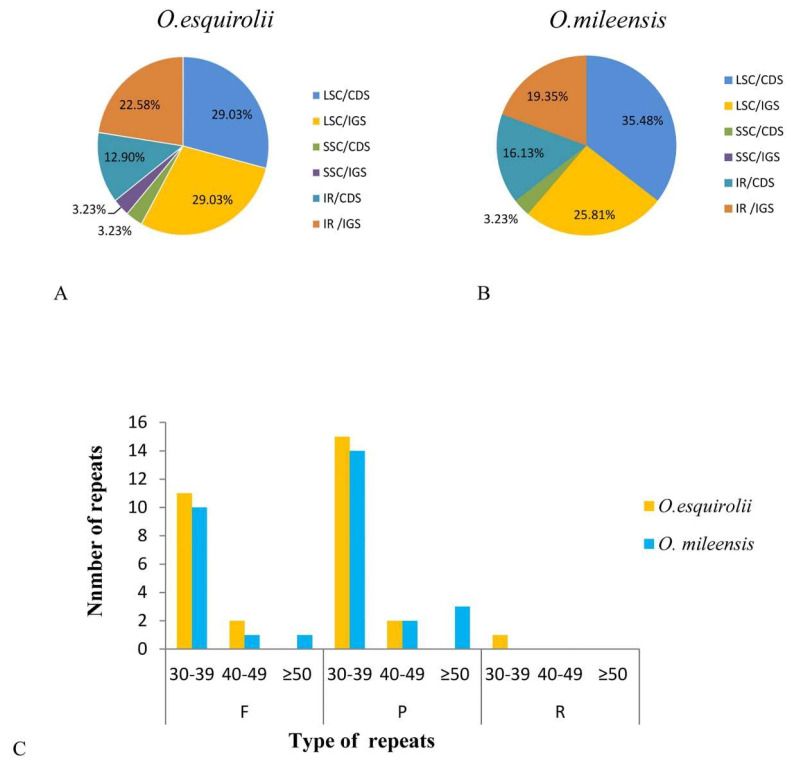
Repeat sequences in the chloroplast genome of *Oreocharis esquirolii* and *O. mileensis*. (**A**,**B**) Percentages of repeats in LSC, IR, and SSC regions; (**C**) Numbers of repeat types detected (F: forward, P: palindrome, R: reverse).

Thirty-one repeat sequences were identified in both cp genomes of *Oreocharis mileensis* and *O. esquirolii*. In *O. esquirolii*, 13 (41.94%) forward repeats, 17 (54.84%) palindromic repeats, and one (3.23%) reverse repeats were identified. Similarly, in *O. mileensis*, palindromic repeats (19, 61.29%) are the most frequent, followed by forward repeats (12, 38.71%). However, none reverse repeats were identified in *O. mileensis* (Figure 4C, Table S4). Additionally, in the cp genome of *O. esquirolii*, the repeat sequence length ranged from 30 bp to 56 bp, while in *O. mileensis*, the length varied from 30 bp to 137 bp. Further analysis of the percentage of repeats in LSC, SSC and IR regions of *O. esquirolii,* and *O. mileensis* revealed that the LSC contained the largest number of repeats, accounting for 58.06%, and 61.29%, respectively, followed by the IR region with 35.48% and 35.48%, and the SSC region with 6.46% and 3.23% (Figure 4A,B).

### 2.5. Comparisons of Chloroplast Genome among Oreocharis esquirolii and Closely Related Species

**Figure 5:**
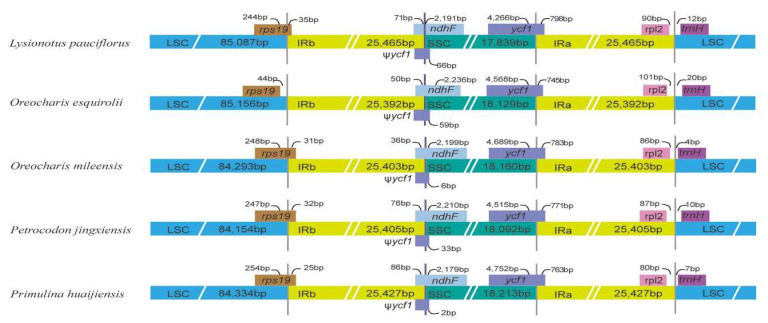
Comparisons of LSC, SSC, and IR border regions among five chloroplast genomes of Gesneriaceae.

Expansion and contraction of the IR region, contributing to variation of cp genome size, plays a crucial role in the evolution of plants [42,43]. Junctions between single copy regions and IR regions among closely related species of *Lysionotus pauciflorus*, *Petrocodon jingxiensis*, *Primulina huaijiensis*, *Oreocharis esquirolii*, and *O. mileensis* were compared in this study. These genomes showed a bit variances at the junctions, but the general gene structures, contents, and orientations were the same. The LSC/IRb junction had expanded to *rps19* gene in four species (*Lysionotus pauciflorus*, 35 bp, *Oreocharis mileensis*, 31 bp, *Petrocodon jingxiensis*, 32 bp, and *Primulina huaijiensis*, 25 bp). However, in *O. esquirolii*, the *rps19* gene did not span the LSC/IRb junction (44 bp away from the junction), suggesting that the IR regions of *O. esquirolii* underwent significant contraction compared with the other four species. This phenomenon was also observed in *Streptocarpus* [31]. A pseudogenized *ycf1* occurred at the IRb/SSC junctions in all species as a result of the extension of SSC/IRa junction into the *ycf1* gene, with variable extensions of the gene into the SSC region observed in the five species. In contrast, *ycf1* was mainly located in the SSC region ranging from 4752 bp to 4266 bp. An overlap of Ψ*ycf1* and *ndhF* genes was observed in all five species: *Lysionotus pauciflorus* (137 bp), *O. mileensis* (42 bp), *Primulina huaijiensis* (88 bp), *O. esquirolii* (109 bp), and *Petrocodon jingxiensis* (109 bp) (Figure 5).

**Figure 6:**
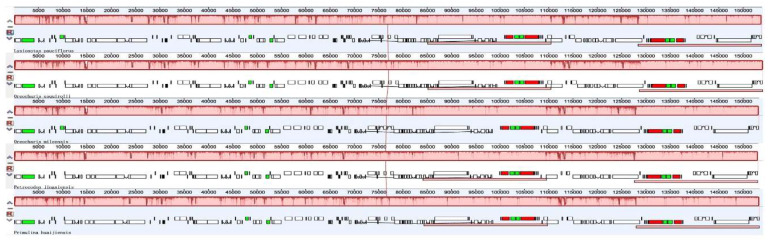
Mauve multiple alignment of five chloroplast genomes of Gesneriaceae, with *Oreocharis esquirolii* as the reference.

Mauve was used to check for possible rearrangements within the cp genomes of five species (*Lysionotus pauciflorus*, *Orecharis esquirolii*, *O. mileensis*, *Petrocodon jingxiensis*, and *Primulina huaijiensis*). The results indicated that the organization of the five Gesneriaceae cp genome was highly conserved, without translocations or inversions detected (Figure 6).

**Figure 7:**
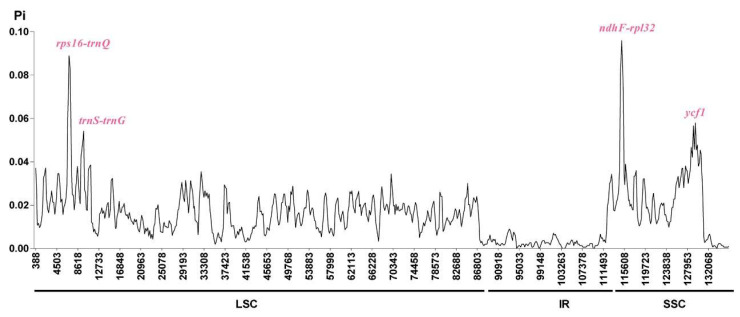
Nucleotide diversity (Pi) in the complete chloroplast genomes of five species of Gesneriaceae. Sliding window analysis with a window length of 600 bp and a step size of 200 bp.

A sliding window analysis was used to estimate the level of variation across regions in the five Gesneriaceae cp genomes. The nucleotide diversity (Pi) values ranged from 0.00000 to 0.09606, with a mean of 0.01381. All highly divergent sequences were restricted to the single copy (SC) regions, with the highest peak occurring in the SSC region. Four hyper-variable regions were identified with nucleotide diversity values higher than 0.05, of which three were intergenic spacers (*rps16-trnQ*, *trnS-trnG*, and *ndhF-rpl32*), and the remaining one was *ycf1* gene (Figure 7). Generally, the intergenic regions exhibit higher nucleotide diversity than the coding regions. As expected, of the four hypervariable regions detected in five Gesneriaceae cp genomes, three were in intergenic regions, while only one in genic region. Similar result was also found in recent cp genome analysis [31,43]. Although not commonly used because of large number of primer pairs needed to sequence the entire region, as a hypervariable gene detected here, *ycf1* could be regarded as a potential marker in phylogenetic analysis of Gesneriaceae, and it have been demonstrated to be effective in Orchidaceae and Lamiaceae [44,45].

**Figure 8:**
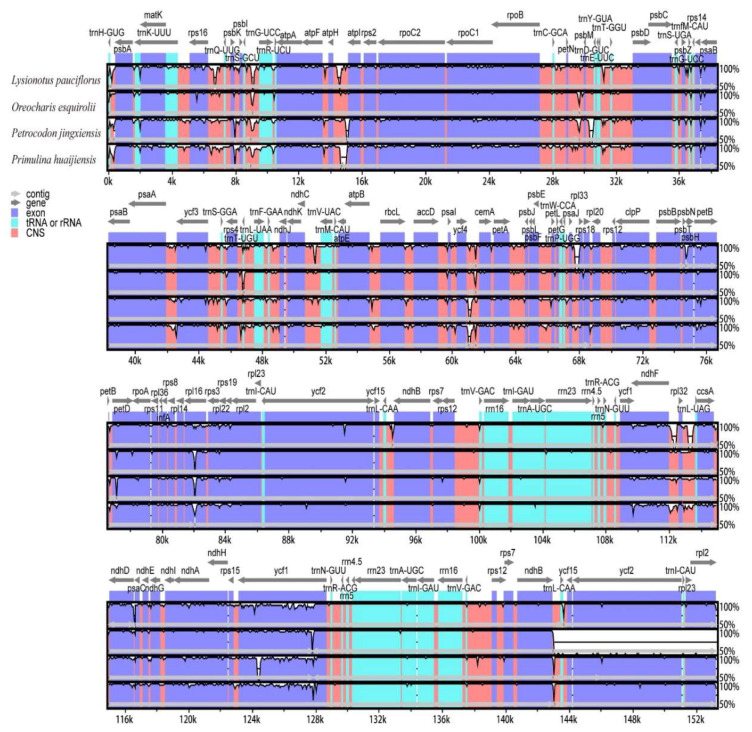
Visualization of genome alignment of five chloroplast genomes of Gesneriaceae using *Oreocharis mileensis* as reference.

The pairwise cp genomic alignment between *O. esquirolii* and its closely related species was analyzed using mVISTA with the annotation of *O. mileensis* as a reference. Results showed that IR regions were found to be more conserved than the single copy regions, so were genic regions, coding regions, and exons compared with intergenic regions, non-coding and introns. Highly divergent regions among the five species of cp genomes were mainly located in the intergenic spacers, such as *trnH^-GUG^-psbA*, *rps16-trnQ^-UUG^*, *atpH-atpI*, *trnL^-UAG^-ccsA*, and *ycf4-cemA*, and few (*rpl16* and *ycf1*) were distributed in protein-coding regions (Figure 8). These regions can provide phylogenetic information as well as serve as unique barcodes for DNA. 

### 2.6. Phylogenetic Position of Oreocharis esquirolii

**Figure 9:**
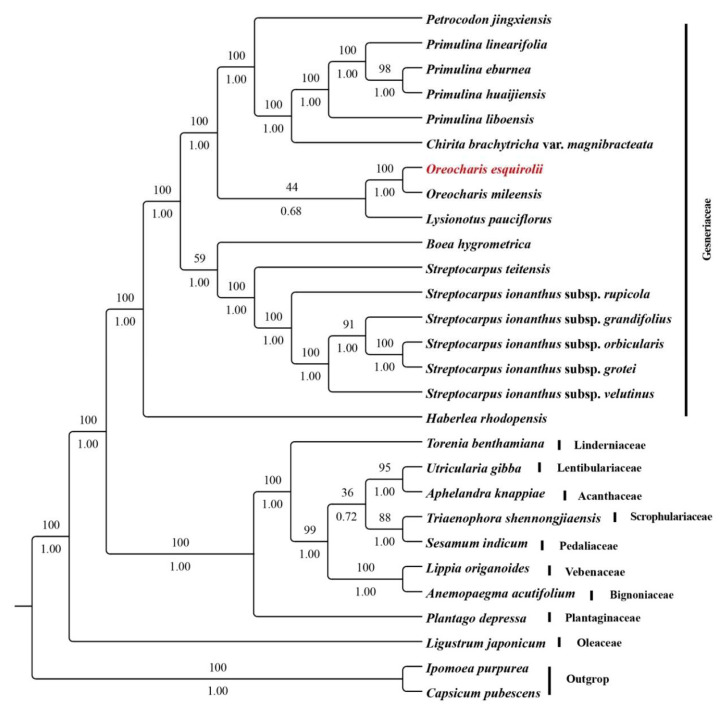
Phylogenetic relationships of 28 species based on complete chloroplast genome sequence. ML bootstrap values are given above branches and posterior probabilities are indicated below.

Based on whole cp genome sequences of 26 taxa within Lamiales, the phylogenetic relationship of Gesneriaceae was inferred using Bayesian inference (BI) and maximum likelihood (ML) analyses. As topology of BI and ML trees were identical, the two trees were combined with addition of bootstrap values of ML and posterior probabilities values of BI. Phylogenetic results showed Gesneriaceae was monophyletic, and *O. esquirolii* grouped with *O. mileensis* (Figure 9). As bearing actinomorphic corolla, Wang [13] transferred *O. esquirolii* to *Thamnocharis esquirolii*. Together with other genera such as *Bournea*, *Tengia*, and *Conandron*, *Thamnocharis* was classed into tribe Ramondieae that is sometimes considered to be primitive in Gesneriaceae [2]. However, phylogenetic analysis showed that actinomorphic genera are scattered over clades with zygomorphic corolla, and hypothesized that flora actinomorphy has evolved in a convergent manner [13]. In addition, phylogenetic studies also indicated that *Oreocharis* is non-monophyletic with several genera including *Thamnocharis* embedded [3,4,46], and finally, Möller et al. [4] regarded *Thamnocharis esquirolii* as a synonym of *Oreocharis esquirolii*. Although the sampling is very limited in our analysis, the sister relationship between *Oreocharis esquirolii* and *O. mileensis* support resurrection of the name *Oreocharis esquirolii* from *Thamnocharis esquirolii*. 

## 3. Materials and Methods

### 3.1. Plant Material, DNA Extraction, Sequencing, and Assembly

Young leaves of *Oreocharis esquirolii* were collected from Longtoudashan Natural Reverse, Zhenfeng, Guizhou, Southwestern China, and were put into silica gel to preserve. Total genomic DNA was extracted from about 100 mg of dried leaf material according to a modified CTAB method [47]. DNA integrity was assessed by electrophoresis on a 1% agarose gel and its concentration and yield was determined and calculated with Qubit. The DNA sample meeting the requirements of sequencing was sent to the BGI-Wuhan and Illumina HiSeq 2500 platform was used for sequencing. After filtering the low-quality data and adaptors, clean data were obtained. Then, GetOrganelle [48], a fast toolkit for accurate *de novo* assembly of organelle genomes which was jointly completed by SPAdes [49], Bowtie2 [50], and BLAST+ [51], was used to assemble the cp genome of *O. esquirolii* with *O. mileenis* (MK342624) [28] as a reference. Assembly graph was visualized using Bandage v.8.0 [52] and then a whole circular cp genome was generated.

### 3.2. Genome Annotation and Sequence Submission

The cp genome was annotated using program PGA [53] with *Oreocharis mileensis* [28] as a reference, then coupled with manual adjustment using Geneious v.10.1.3 [54]. MEGA 6.06 [55] was used to analyze AT and GC contents. Finally, the circular genome map was generated with OGDRAW v.1.2 [56] and submitted to NCBI GenBank under Accession Number MT612436.

### 3.3. Codon Usage, Repetitive Sequence, and SSR Analysis

The codon usage frequency was calculated based on protein-coding genes using CodonW 1.4.2. [57]. REPuter [58] was used to identify repeat sequences, including direct (forward), inverted (palindromic), complement, and reverse repeats. The repeat sizes were limited to a minimum of 30 bp and a maximum of 300, with sequence identities greater than 90% (Hamming distance of 3). MISA [59], an SSR motif scanning tool written in Perl, was adopted to detect SSRs. The minimum thresholds were set to 10 repeat units for mononucleotide SSRs, four repeat units for dinucleotide and trinucleotide SSRs, and three repeat units for tetranucleotide, pentanucleotide, and hexanucleotide SSRs [60].

### 3.4. Genome Comparison

Based on previous phylogenetic results together with the current reported data, five Gesneriaceae cp genomes (*Lysionotus pauciflorus*, *Petrocodon jingxiensis*, *Primulina huaijiensis*, *Oreocharis mileensis*, and *O. esquirolii*) were selected for comparative analysis. To explore the expansion and contraction of IR regions of *Oreocharis esquirolii*, comparison of boundaries between IRs and single copy regions was performed in Geneious v.10.1.3 [54]. The mVISTA [61] was used to assess the similarity among the five cp genomes, and the default parameters were utilized to align the cp genomes in Shuffle-LAGAN mode. Chloroplast genome sequence alignment was carried out with the Mauve program [62] to check the gene order and sequence variations. Sliding window analysis of nucleotide variability in the cp genome was conducted using DnaSP [63]. The step size was set to 200 bp, with a 600 bp window length.

### 3.5. Phylogenetic Analyses

To explore the phylogenetic position of *Oreocharis esquirolii* among the limited number of species available across Gesneriaceae, complete cp genomes of 26 species within Lamiales were selected to conduct analyses, using *Ipomoea purpurea* and *Capsicum pubescens* from Solanales as outgroups [64,65] (Table S5). Multiple sequence alignment of cp genome sequences were performed using MAFFT [66], and poorly aligned positions and regions with a too-high divergence were excluded from the alignment using Gblocks v0.91 [67]. Bayesian inference (BI) and Maximum likelihood (ML) methods were adopted for phylogenetic analyses. ML analysis was performed using RAxML–HPC2 on XSEDE v.8.2.12 as implemented on the CIPRES Science Gateway (http://www.phylo.org/) [68] under the GTRGAMMA model. Bootstrap iteration (–#|–N) was set to 1000, and other parameters followed default settings. BI analysis was performed in MrBayes v3.2.6 [69] as implemented in PhyloSuite [70] with the ModelFinder [71] used to select the best model. Under the Akaike information criterion (AIC), the GTR+F+I+G4 model was selected for the data matrix. The Markov Chain Monte Carlo (MCMC) algorithm was calculated for 2,000,000 generations with two parallel searches using four chains, each starting with a random tree. The convergence was reached with the average standard deviation of split frequencies (ASDFs) following 0.01. Trees were sampled at every 1000 generations with the first 25% discarded as burn-in, and the remaining trees were used to construct majority-rule consensus trees.

## 4. Conclusions

*Oreocharis esquirolii*, also known as *Thamnocharis esquirolii*, is categorized under IUCN criteria as vulnerable. We assembled and characterized the complete cp genome of *O. esquirolii* for the first time. The cp genome features of *O. esquirolii* were similar to other reported species of Gesneriaceae concerning gene content, order, and orientation. SSRs analysis supports the view that cp SSRs are generally composed of short polyA or polyT, and pentanucleotide and hexanucleotide repeats are rather rare. Comparative analyses revealed that no arrangements occurred in Gesneriaceae, intergenic regions were more variable than coding regions, and some hypervariable regions such as *rps16-trnQ*, *trnS-trnG*, *ndhF-rpl32* and *ycf1* may be applied to address phylogenetic issues of Gesneriaceae. Phylogenetic analysis supported synonymizing *Thamnocharis esquirolii* as *Oreocharis esquirolii*. The complete cp genome sequence will contribute to further studies in molecular identification, genetic diversity, and phylogeny.

## Figures and Tables

**Table 1:** Genes present in chloroplast genome of *Oreocharis esquirolii*.

Category	Gene Group	Gene Names
Photosynthesis	Subunits of ATP synthase	*atpA*, *atpB*, *atpE*, atpF *, *atpI*, *atpH*
	Subunits of NADH dehydrogenase	ndhA *, ndhB * (×2), *ndhC*, *ndhD*, *ndhE*, *ndhF*, *ndhG*, *ndhH*, *ndhI*, *ndhJ*, *ndhK,*
	Subunits of cytochrome	*petA*, *petB*, *petD*, *petG*, *petL*, *petN*
	Subunits of photosystem I	*psaA*, *psaB*, *psaC, psaJ*, *psaI*
	Subunits of photosystem II	*psbA*, *psbB*, *psbC*, *psbD*, *psbE*, *psbH*, *psbK*, *psbN*, *psbJ*, *psbF*, *psbL*, *psbI*, *psbM*, *psbT*, *psbZ*
	Subunit of rubisco	*rbcL*
Other genes	Subunit of Acetyl-CoA-carboxylase	*accD*
	c-type cytochrome synthesis gene	*ccsA*
	Envelop membrane protein	*cemA*
	Protease	*clpP* **
	Translational initiation	*infA*
	Maturase	*matK*
Self-replication	Large subunit of ribosome	rpl2 * (×2), *rpl14*, rpl16 *, *rpl20*, *rpl22*, *rpl23* (×2)*, rpl32*, *rpl33*, *rpl36*
	DNA dependent RNA polymerase	*rpoA*, *rpoC2*, *rpoB*, *rpoC1*
	Small subunit of ribosome	rps12 ** (×2), *rps2*, *rps3*, *rps4*, *rps7* (×2), *rps8*, *rps11*, *rps14*, *rps15*, rps16 *, *rps18*, *rps19*
	rRNA Genes	*rrn4.5* (×2), *rrn5* (×2), *rrn16* (×2), *rrn23* (×2)
	tRNA Genes	trnK-UUU *, trnI-GAU * (×2), trnA-UGC * (×2), trnV-UAC *, trnL-UAA *, *trnS-UGA*, *trnS-GCU*, *trnS-GGA*, *trnY-GUA*, *trnL-CAA* (×2), *trnL-UAG*, *trnL-GAG*, *trnM-CAU*, *trnR-ACG* (×2), *trnP-UGG*, *trnW-CCA*, *trnD-GUC*, *trnH-GUG*, *trnF-GAA*, *trnT-UGU*, *trnE-UUC*, *trnN-GUU* (×2), *trnV-GAC* (×2), *trnT-GGU*, *trnQ-UUG*, *trnR-UCU*, *trnG-GCC*, *trnC-GCA*, *trnI-CAU (×2)*, *trnfM-CAU*
Unknown function	Conserved open reading frames	*ycf1* (×2, ψ), *ycf2 (×2)*, ycf3 **, *ycf4*, *ycf15* (×2)

(×2) gene in two copies, * gene which contains one intron, ** gene which contains two introns, ψ one of two duplicated genes is a pseudogene.

**Table 2:** Length of exons and introns within intron-containing genes in the chloroplast genome of *Oreocharis esquirolii*.

Gene	Region	Exon1 (bp)	Intron1 (bp)	Exon2 (bp)	Intron2 (bp)	Exon3 (bp)
*atpF*	LSC	144	707	411		
*ndhA*	SSC	552	1062	540		
*ndhB*	IR	777	679	756		
*rpl2*	IR	390	673	435		
*rpl16*	LSC	9	824	399		
*rps16*	LSC	42	921	210		
*clpP*	LSC	69	814	291	644	228
*rpoC1*	LSC	453	812	1611		
*trnA-UGC*	IR	38	807	35		
*trnI-GAU*	IR	37	941	35		
*trnK-UUU*	LSC	37	2497	36		
*trnL-UAA*	LSC	37	476	48		
*trnV-UAC*	LSC	38	586	35		
*ycf3*	LSC	126	692	228	714	153

**Table 3:** AT and GC content in different regions in the chloroplast genome of *Oreocharis esquirolii*.

Region	Length (bp)	A (%)	T (%)	G (%)	C (%)	GC (%)
LSC	85,156	31.54	33.03	17.30	18.13	35.43
SSC	18,129	34.34	34.50	15.00	16.16	31.16
IRA	25,392	28.38	28.41	22.42	20.79	43.21
IRB	25,392	28.38	28.41	22.42	20.79	43.21
CDS	79,650	30.70	31.59	20.10	17.61	37.71
Total genome	154,069	30.83	31.67	18.45	19.04	37.49

## Supplementary Materials

The following are available online at https://www.mdpi.com/2223-7747/9/12/1692/s1, Table S1: Comparison of the features of *Oreocharis esquirolii* with other Gesneriaceae chloroplast genomes. Table S2: Comparative analysis of chloroplast codon usage bias of *Oreocharis esquirolii*. Table S3: Distribution of simple sequence repeats (SSRs) loci in the chloroplast genome *Oreocharis esquirolii* and *O. mileensis*. Table S4: List of repeated sequences and their locations in chloroplast genome of *Oreocharis esquirolii* and *O. mileensis*. Table S5: Taxa used in phylogenetic analysis in this study.
